# Immobilization of Lipase from *Thermomyces Lanuginosus* and Its Glycerolysis Ability in Diacylglycerol Preparation

**DOI:** 10.3390/molecules29174141

**Published:** 2024-08-31

**Authors:** Rui Xie, Yee-Ying Lee, Pengkai Xie, Chin-Ping Tan, Yong Wang, Zhen Zhang

**Affiliations:** 1 JNU-UPM International Joint Laboratory on Plant Oil Processing and Safety, Department of Food Science and Engineering, Jinan University, Guangzhou 510632, China; xierui0911@163.com (R.X.); xiepk@stu2023.jnu.edu.cn (P.X.); twyong@jnu.edu.cn (Y.W.); 2 School of Science, Monash University Malaysia, Bandar Sunway, Subang Jaya 47500, Selangor, Malaysia; lee.yeeying@monash.edu; 3 Department of Food Technology, Faculty of Food Science and Technology, University Putra Malaysia (UPM), Serdang 43400, Selangor, Malaysia; tancp@upm.edu.my

**Keywords:** biocatalysis, catalytic stability, enzyme stability, lipase immobilization

## Abstract

In the glycerolysis process for diacylglycerol (DAG) preparation, free lipases suffer from poor stability and the inability to be reused. To address this, a cost-effective immobilized lipase preparation was developed by cross-linking macroporous resin with poly (ethylene glycol) diglycidyl ether (PEGDGE) followed by lipase adsorption. The selected immobilization conditions were identified as pH 7.0, 35 °C, cross-linking agent concentration 2.0%, cross-linking time 4 h, lipase amount 5 mg/g of support, and adsorption time 4 h. Enzymatic properties of the immobilized lipase were analyzed, revealing enhanced pH stability, thermal stability, storage stability, and operational stability post-immobilization. The conditions for immobilized enzyme-catalyzed glycerolysis to produce DAG were selected, demonstrating the broad applicability of the immobilized lipase. The immobilized lipase catalyzed glycerolysis reactions using various oils as substrates, with DAG content in the products ranging between 35 and 45%, demonstrating broad applicability. Additionally, the changes during the repeated use of the immobilized lipase were characterized, showing that mechanical damage, lipase leakage, and alterations in the secondary structure of the lipase protein contributed to the decline in catalytic activity over time. These findings provide valuable insights for the industrial application of lipase.

## 1. Introduction

In recent years, with rising living standards and shifts in dietary habits, the global prevalence of obesity and cardiovascular diseases associated with excessive fat intake have become increasingly concerning. In response, there has been a surge of interest in functional oils. Diacylglycerol (DAG), a functional lipid, is a naturally occurring minor component in oils, comprising a glycerol backbone bonded to two fatty acid molecules. DAG exists in three isomeric forms: sn-1,2 DAG, sn-1,3 DAG, and sn-2,3 DAG [1]. Studies have shown that DAG consumption can significantly reduce visceral fat and total cholesterol levels, providing multiple health benefits, including the inhibition of fat accumulation, the regulation of blood lipid levels, and the prevention of diabetes, thus making it highly valuable for human health [2]. Moreover, DAG exhibits excellent emulsifying and moisturizing properties, contributing to its widespread use in the cosmetics industry. Its applications in biodiesel production and chemical synthesis further underscore its importance in the renewable energy and chemical industries. DAG holds immense potential and value in enhancing food safety, promoting health, and advancing green energy and biotechnology research. The preparation of DAG primarily involves chemical and enzymatic methods. Among these, enzymatic glycerolysis has emerged as a key focus of recent research and application due to its efficiency and environmental benefits [3]. Enzymatic glycerolysis employs specific lipases to catalyze the reaction between triacylglycerol (TAG) and glycerol under mild conditions, offering advantages such as high selectivity, reaction at low temperatures and neutral pH, and the absence of harmful by-products. This method not only improves the production efficiency of DAG but also holds significant promise for green energy and biotechnology research [4,5].

Lipase (E.C. 3.1.1.3, triacylglycerol acylhydrolase) is a crucial enzyme widely utilized for catalyzing the hydrolysis of insoluble triacylglycerol at the oil–water interface into DAG, monoacylglycerol (MAG), glycerol, and free fatty acid (FFA) [6,7]. Its extensive applications span the food, pharmaceutical, cosmetic, and biodiesel production industries, garnering significant attention [8,9]. Microbial lipases, in particular, serve as biocatalysts in various non-aqueous reactions such as esterification, transesterification, interesterification, acidolysis, alcoholysis, and aminolysis, owing to their diversity, substrate specificity, catalytic properties, and high stability in reaction media [10]. The majority of lipases employed as catalysts in green reactions are derived from microbial sources, including *Aspergillus niger*, *Burkholderia cepacia*, *Rhizomucor miehei*, and *Thermomyces lanuginosus*, due to their ease of production via fermentation and straightforward purification procedures [11]. *Thermomyces lanuginosus* lipase (TLL) is notably basophilic and thermostable, comprising a single-chain protein of 269 amino acids, with a molecular weight of 31.7 kDa, an isoelectric point of 4.4, and molecular dimensions of 35 Å × 45 Å × 50 Å [12]. TLL exhibits high catalytic activity, strict enantioselectivity, and broad specificity, enabling it to catalyze a wide range of reactions involving both natural and synthetic substrates. Consequently, it is extensively used in various industries, from biodiesel production to fine chemicals, particularly in oil and fat modification. However, the practical application of lipases is often hindered by their relatively low stability and challenges in recycling and reuse [13]. To address these issues, enzyme immobilization has emerged as a crucial strategy [14].

Enzyme immobilization involves anchoring the enzyme molecule onto a solid support to improve its stability and enable its recovery and reuse. The methods of immobilization include adsorption, cross-linking, covalent bonding, and encapsulation, among others [15,16]. Physical adsorption is a technique for the reversible immobilization of lipase, relying on weak, nonspecific forces such as van der Waals forces, hydrophobic interactions, hydrogen bonds, and ionic interactions to adhere the lipase to the support surface. This method’s primary advantage is that it maintains the immobilized enzyme’s structural conformation and high activity. However, it has limitations. Physical adsorption is sensitive to variations in pH, temperature, and buffer ionic strength. Additionally, the weak physical bonds may not adequately secure the lipase on the support, leading to enzyme leaching [17]. Lipases possess a helical lid covering their active sites, which typically exhibit full catalytic activity only upon interfacial activation. During adsorption at hydrophobic/hydrophilic interfaces, the conformation of this lid changes to an “open” active state, thereby enhancing the enzyme’s activity. However, interactions of the biocatalyst with detergent-like substrates or products can weaken its adsorption strength and lead to enzyme desorption. In the production of DAG, the presence of free fatty acids, DAG, and MAG may remove lipases from hydrophobic support, adversely affecting their catalytic capabilities. Cross-linking is a frequently employed technique for enzyme immobilization. In this method, enzymes are attached to a support matrix via covalent bonds created by cross-linking agents [18]. This approach enhances various aspects of enzyme stability, such as temperature, pH, and operational stability, by providing a sturdy support structure that preserves enzyme activity. Cross-linking minimizes enzyme leakage, thereby improving reusability and cost-effectiveness, making it highly suitable for industrial uses. Nonetheless, there are some disadvantages associated with cross-linking. The process can modify the enzyme’s active site, potentially diminishing its catalytic activity. Additionally, it is typically more complex and time-consuming than other immobilization techniques, necessitating careful optimization to achieve the appropriate degree of cross-linking without negatively affecting enzyme performance [19,20]. Therefore, the combination of adsorption and cross-linking methods was considered to enhance the stability of immobilized enzymes to a certain extent. This method involved an initial cross-linking step, where the support material was treated with a cross-linking agent, followed by the adsorption of the enzyme onto the pre-treated support. Additionally, the method offers operational advantages, such as easier control and the optimization of reaction conditions. The immobilized enzyme could be readily separated from the reaction mixture, facilitating better control over the reaction parameters and improving overall process efficiency. This study focused on the immobilization of lipase using this method, exploring its potential benefits and applications.

The choice of support material plays a crucial role in the immobilization process. An optimal support should offer a high surface area for enzyme attachment, demonstrate biocompatibility, and preserve the enzyme’s activity and stability. Various materials have been investigated for this purpose, including organic polymers, inorganic supports, and hybrid composites [21]. LX-201A, a styrene-based non-polar weakly polar polymer, is hydrophobic and can mitigate the decrease in catalytic activity caused by glycerol at the enzyme–substrate interface [20]. It creates conducive conditions for the interaction between lipase and linseed oil, making it an appropriate choice for immobilization. Therefore, LX-201A was chosen as the support for immobilization in this study.

In this study, lipase CN-TL from *Thermomyces lanuginosus* was immobilized using macroporous resin LX-201A and poly (ethylene glycol) diglycidyl ether (PEGDGE) via a method involving cross-linking the resin with the cross-linking agent followed by enzyme adsorption. The cross-linking and adsorption conditions were selected, and the enzymatic properties were analyzed. Subsequently, the conditions for immobilized enzyme-catalyzed glycerolysis to produce DAG were selected, and different types of oils were used as substrates to verify the applicability of the immobilized lipase. Finally, the changes during the repeated use of the immobilized lipase were characterized. This research provides a reference for the application of immobilized lipase in oil processing.

## 2. Results and Discussion

### 2.1. Selection of Immobilization Conditions

**Figure 1:**
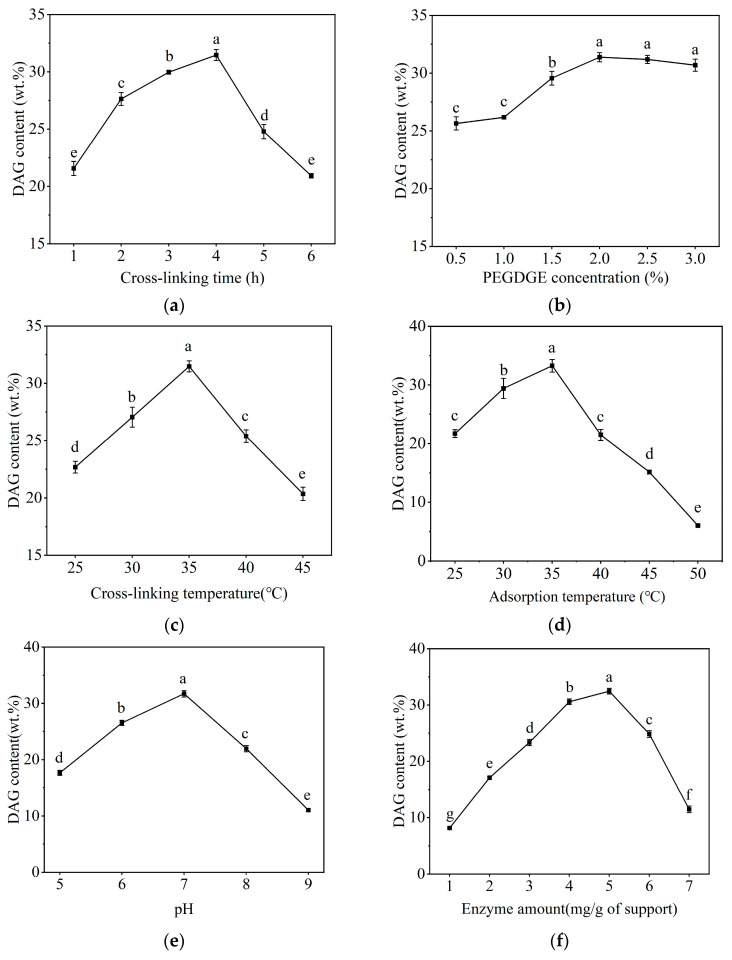

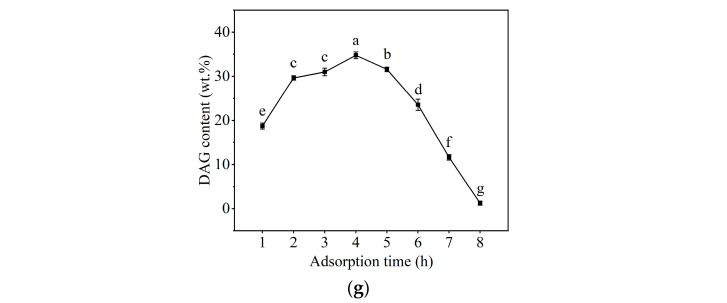
Selection of the preparation conditions for TL-PEGDGE-LX: (**a**) cross-linking time; (**b**) cross-linking agent concentration; (**c**) cross-linking temperature; (**d**) adsorption temperature; (**e**) pH value; (**f**) enzyme loading; (**g**) reaction time.

To acquire immobilized lipase suitable for catalyzing glycerolysis, critical factors such as cross-linking time, cross-linking agent concentration, and cross-linking temperature were selected during the immobilization process, as shown in Figure 1a. A trend of increased DAG content followed by a decrease was observed with the prolongation of cross-linking time. The glycerolysis process yielded the highest DAG content after 4 h of cross-linking, attributable to the enhanced structural strength of the immobilized lipase from the extended cross-linking time. Nonetheless, the cross-linking agent may adversely affect the amino acids at the lipase’s active site, thereby affecting the lipase’s catalytic ability [22]. The content of DAG in the glycerolysis product catalyzed by immobilized lipase increased initially and then decreased with the rise in cross-linking agent concentration, reaching its peak at a concentration of 2.0%. A moderate increase in the cross-linking agent concentration served to stabilize the immobilized lipase. However, overly strong interactions could compromise the conformation of the lipase. Furthermore, excessively high concentrations may lead to intermolecular and intramolecular cross-linking of lipase molecules, or even cross-linking with the amino acid groups at the lipase’s active site, resulting in diminished enzymatic activity [23]. Cross-linking temperature also significantly impacts the catalytic ability of lipase for glycerolysis. The cross-linking process achieved the maximum DAG content at 35 °C, and the catalytic ability for glycerolysis decreased significantly with a further increase in cross-linking temperature, suggesting that elevated temperatures may compromise the structure of the lipase and thus reduce its activity. Additionally, higher temperatures accelerated molecular movement and led to a decrease in lipase activity due to strong interaction with the cross-linking agents.

The effect of adsorption temperature, pH value, lipase loading, and adsorption duration on lipase catalytic activity is shown in Figure 1d–g. The product’s DAG content increased with temperature, reaching an optimum at 35 °C. This increase is attributed to intensified thermal molecular motions that enhanced the substrate molecules’ engagement with the lipase’s active site. However, beyond this temperature, the DAG content declined as excessively high temperatures may have induced conformational alterations or even the inactivation of the lipase. Similarly, pH significantly influenced the enzyme’s activity (Figure 1e). The highest DAG content in the glycerolysis product was observed at pH 7, where the pH environment maintained the lipase’s native conformation. Deviations from this pH can lead to structural changes that diminish lipase efficiency or cause inactivation. The concentration of lipase is also an important factor affecting enzymatic catalytic ability. With an increase in lipase concentration, the content of DAG in the glycerolysis product catalyzed by immobilized lipase showed a trend of gradual increase followed by a decrease, reaching its zenith at a lipase loading of 5 mg/g (resin). Generally, an elevated lipase concentration within a certain range can enhance catalytic activity due to the increased efficient catalytic sites. Nevertheless, excessively high levels of lipase may instigate aggregation and impede effective reaction mixing, limiting mass transfer, which can lead to uneven lipase adsorption and a subsequent reduction in lipase catalytic ability, impacting the interaction between lipase molecules and their substrates [24]. Additionally, the DAG content in the products from the glycerolysis reactions, catalyzed by the immobilized lipase, showed a progressive increase as the immobilization time was extended, reaching its maximum at 4 h (Figure 1g). Prolonging the adsorption duration served to increase the amount of lipase that was immobilized, thereby facilitating the lipase-catalyzed glycerolysis reaction. However, a catalytic decline was observed when the immobilization exceeded 4 h, a phenomenon potentially linked to excessive lipase immobilization, which resulted in spatial hindrance that blocked some of the immobilized lipase’s active sites.

### 2.2. Stability of Immobilized Lipase

**Figure 2:**
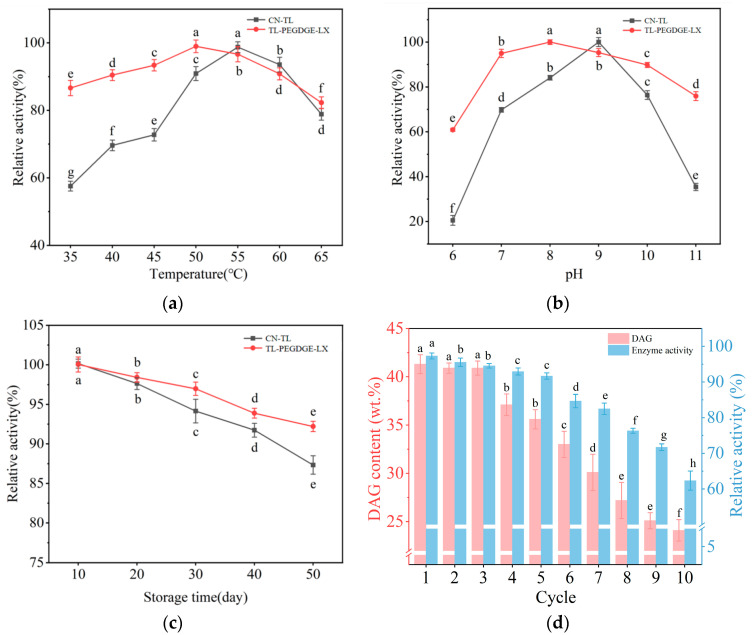
Stability of the immobilized lipase TL-PEGDGE-LX and the free lipase CN-TL: (**a**) thermal stability; (**b**) pH stability; (**c**) storage stability; (**d**) reusability.

The thermal stability of lipase is a critical indicator for assessing its application value. The optimal temperature of lipase is essential in determining the most suitable conditions for its application. The effect of reaction temperature on the activity of both free lipase CN-TL and immobilized lipase TL-PEDGE-LX was investigated within the range of 35–65 °C, as illustrated in Figure 2a. It was observed that the optimal temperature for lipase was 55 °C, while at 35 °C, its activity decreased to 47.55% of this maximum. In contrast, immobilized lipase maintained over 80% of its maximal activity across a broad temperature range from 35 °C to 65 °C—a significantly wider range of thermal stability compared to the 50–60 °C range for free lipase, demonstrating enhanced thermal stability. This increased stability can be attributed to the pore structure of the resin, which provided a protective barrier for the lipase, preventing conformational changes at high temperatures. Additionally, the cross-linking interactions between the resin and lipase molecules inhibited the stretching deformation of the lipase molecules, thereby reinforcing the main chain of the lipase and enhancing its resistance to high-temperature-induced degradation. Consequently, this structural reinforcement allows the lipase to maintain its catalytic efficiency at elevated temperatures [25].

pH is a crucial factor affecting catalytic activity during the catalytic process. The catalytic activities of free lipase CN-TL and immobilized lipase TL-PEDGE-LX at different pH levels are shown in Figure 2b. CN-TL exhibited maximum activity at pH 9.0, maintaining over 80% of its maximum activity within the pH range of 8–9. However, at pH 6 and pH 11, there was a significant decrease in activity to 20.52% and 35.42% of its maximum activity, respectively. In contrast, the immobilized lipase demonstrated superior pH stability, maintaining over 80% of its maximum activity across a pH range of 6–10. The enhanced stability of the immobilized lipase can be attributed to the interactions between lipases and the support surface during the crosslinking adsorption process, which stabilized their tertiary structures. Furthermore, this process reduced interactions between lipase and the interface, reducing lipase dissociation and thereby enhancing the pH stability of the immobilized lipase. Additionally, the optimal pH of the immobilized lipase was pH 8, lower than that of the free lipase. This phenomenon may be explained by the formation of a polymer network by PEGDGE in water, where PEG chains surround the lipase, altering the activity of water molecules around the lipase’s active site. This results in a slightly higher pH value in the microenvironment of the active site compared to that in the external solution [26].

The storage stability of immobilized lipase is essential for industrial applications. This study evaluated the storage stability of both free and immobilized lipases through hydrolytic activity assays after storage at 4 °C for different periods. As shown in Figure 2c, with the extension of storage time, both free and immobilized lipases exhibited a decline in activity. After 50 days of storage, the activity of the free lipase decreased by 12.56%, whereas the activity of the immobilized lipase showed a smaller reduction of 8.70%, indicating enhanced storage stability. This improved stability of the immobilized lipase can be attributed to the stronger interaction between the support and the lipase, the protective effect of the resin, and the cross-linking effect of the PEGDGE, which collectively enhanced the lipase’s adaptability to environmental changes in temperature and moisture.

In addition, the sn-1,3 specificity of TL-PEGDGE-LX was examined. Under identical enzyme activity conditions, TL-PEGDGE-LX and CN-TL were used to catalyze glycerolysis reactions using various oils as substrates. According to the method described by Li et al., the regional specificity of the lipase was evaluated by the content of sn-1,2 DAG and sn-1,3 DAG in the products, as well as the sn-1,3 DAG/sn-1,2 DAG ratio, as detailed in Table 1 [27]. Lipase CN-TL is a typical sn-1,3 specific lipase that preferentially selects the 1,3-position of fatty acid ester bonds in catalyzing glycerolysis reactions, thereby generating sn-1,3 DAG. When using various oil substrates, including olive, canola, peanut, corn, and soybean oils and so on, the sn-1,3 DAG content in the glycerolysis products of TL-PEGDGE-LX was comparable to that catalyzed by CN-TL, confirming the retained specificity of the immobilized lipase.

The repeated usability of the immobilized lipases served as a critical metric for evaluating their application value. Figure 2d illustrates the changes in the immobilized lipase activity over ten successive reactions and the corresponding variations in DAG content in the glycerolysis products. Following five cycles, the immobilized lipase retained over 90% of its initial activity, with the DAG content in the glycerolysis product at 84.8% of the initial reaction. By the tenth cycle, the enzyme activity had reduced to 62.34% of the original activity, indicating that TL-PEGDGE-LX demonstrates good reusability. The resin’s pore structure provided some protection, aiding in resistance to structural damage caused by high temperatures, thus maintaining a more stable structure and enhancing the operational lifespan and recyclability of the lipase. Additionally, PEGDGE played a role in preventing lipase inactivation in adverse microenvironments and effectively immobilized the lipase on the support, thereby minimizing lipase leakage or loss [28].

### 2.3. Characterization of Immobilized Enzyme During Repeated Use

**Figure 3:**
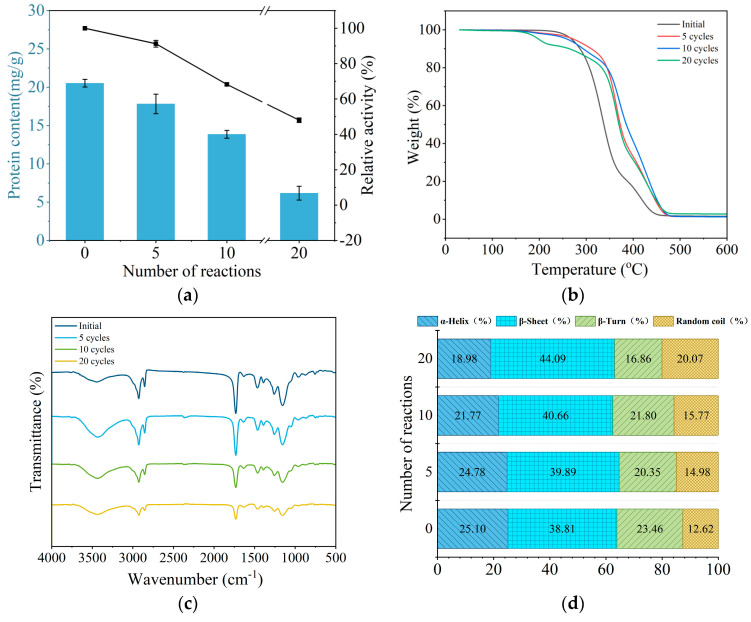

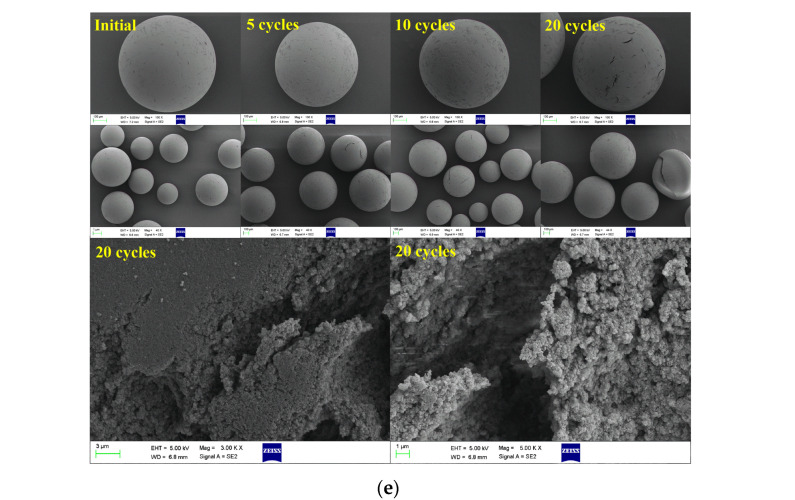
Characterization of immobilized lipase TL-PEDGE-LX during repeated use: (**a**) enzyme activity and protein content; (**b**) thermogravimetric analysis curve; (**c**) Fourier transform infrared spectra; (**d**) secondary protein structure; (**e**) scanning electron microscopy images.

The variations in lipase protein content and activity during the repeated use of immobilized lipase are illustrated in Figure 3a. Both lipase protein content and activity exhibited a declining trend with repeated usage. After five cycles, the enzyme activity retained 91.28% of its initial value, and the protein content retained 88.21%. After twenty cycles, the protein content decreased to 6.17 mg/g, and the enzyme activity dropped by 51.98%. This decline can be attributed to several factors: mechanical wear in the stirring system causing protein loss from both the support and the immobilized lipase due to friction; lipase desorption from the support leading to reduced protein content; and partial lipase degradation influenced by the chemical environment.

The thermogravimetric analysis (TGA) curve of the immobilized lipase is presented in Figure 3b. It can be seen that the more times the immobilized enzyme was used, the faster the initial decline in the TGA curve, suggesting that the enzyme protein may have undergone partial degradation or inactivation, leading to structural instability, and the enzyme may have gradually desorbed from the carrier surface. Additionally, as the number of uses increases, changes in the surface structure or physical properties of the immobilized carrier may occur, weakening the binding force between the carrier and the enzyme. This makes the enzyme more likely to detach or decompose at lower temperatures, causing a faster initial mass decline in the curve. When heated to 600 °C, the residual mass of the immobilized enzyme after 10 uses was slightly lower than after 5 uses, which may have been due to leakage of the enzyme protein during use. However, after 20 uses, the residual mass was higher, possibly due to increased thermal stability of the residues, changes in the carrier structure, or an increase in adsorbed impurities or by-products.

The Fourier Transform Infrared (FTIR) spectra indicated a gradual reduction in the intensity of characteristic absorption peaks with repeated use. The infrared spectrum showed a peak at 1650 cm^−1^, which was attributed to the stretching vibration of C=O and the bending vibration of N-H in the amide I band of the lipase. The peak at 1540 cm^−1^ corresponded to the bending vibrations of C-N and N-H bonds in the amide II band. Additionally, the region between 950 and 1250 cm^−1^ represented the stretching vibrations of C-N, C-O (in primary and secondary alcohols), and the strong bending vibrations of C-H groups [29]. The decline in peak intensity can be attributed to structural changes in the enzyme due to repeated use, affecting specific amino acid residues, and the enzyme may have undergone chemical denaturation or degradation, thus affecting absorption peaks at specific wavelengths. Additionally, physical wear or chemical changes in the immobilization support may have also caused variations in the FTIR spectrum peaks.

Additionally, to investigate the changes in the secondary structure of the immobilized lipase during repeated use, the strongest peak of the amide I band (1600–1700 cm^−1^ region) for TL-PEGDGE-LX was normalized. Established wavenumber ranges were used to quantify the secondary structure composition: β-sheet (1610 cm^−1^ to 1640 cm^−1^), random coil (1640 cm^−1^ to 1650 cm^−1^), α-helix (1650 cm^−1^ to 1660 cm^−1^), and β-turn (1660 cm^−1^ to 1700 cm^−1^) [30]. The changes in the secondary structure of the immobilized lipase during repeated use are illustrated in Figure 3d. After 20 cycles of use, the α-helix content decreased from 25.10% to 18.98%, while the β-sheet content increased from 38.81% to 44.09%, and the β-turns exhibited irregular variations. Repeated use can lead to denaturation or the partial unfolding of the lipase, disrupting the original α-helix structure. Since α-helices are relatively stable components of protein structures, their reduction typically indicates a decline in the overall stability of the lipase, which can directly affect the active site and decrease catalytic efficiency [31]. Additionally, the thermal stability and resistance to the denaturation of the lipase may decrease. Partial unfolding or structural rearrangement of the lipase can lead to the formation of β-sheet structures, which may result from protein refolding or aggregation, further reducing enzyme activity. β-turns, which connect different secondary structural elements within proteins, may undergo rearrangement or formation due to local denaturation or refolding during repeated use. Moreover, the random coil content increased from 12.62% to 20.07%. The increase in random coils is typically due to partial denaturation and loss of ordered secondary structures, indicating that the original stable structure of the protein is compromised, making the lipase more unstable. The increase in random coils indicates that the secondary structure of the lipase no longer maintains its original stability, making the lipase more prone to activity loss. Additionally, the decline in lipase stability reduces its durability in practical applications. It is evident that changes in the secondary structure of lipase proteins are one of the primary factors affecting the reusability of immobilized lipases.

Scanning electron microscopy (SEM) images (Figure 3e) of the immobilized lipase show increased surface roughness, micro-cracks, and pores with repeated use. After twenty cycles, some macro-porous resins exhibit fractures. The glycerolysis reaction, which involves stirring, subjects resin particles to shear forces from mechanical agitation and fluid flow, causing surface and internal structural damage [32]. Prolonged high-temperature operations can further diminish the mechanical strength and stability of the resin, increasing the likelihood of fractures. Additionally, the resin’s pore structure may become clogged with lipases, reaction products, or impurities, reducing pore size and altering surface roughness.

### 2.4. Immobilized Enzyme Catalyzed Glycerolysis for DAG Preparation

**Figure 4:**
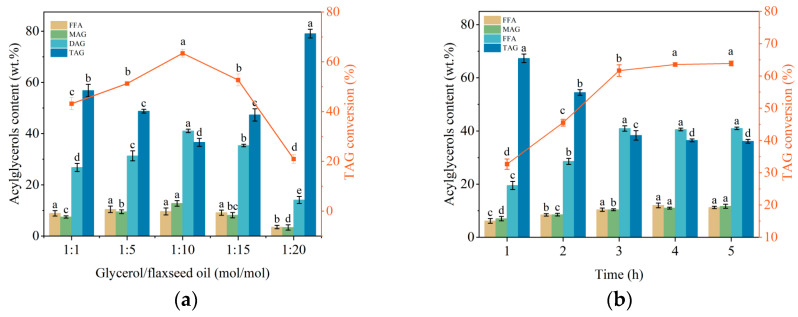

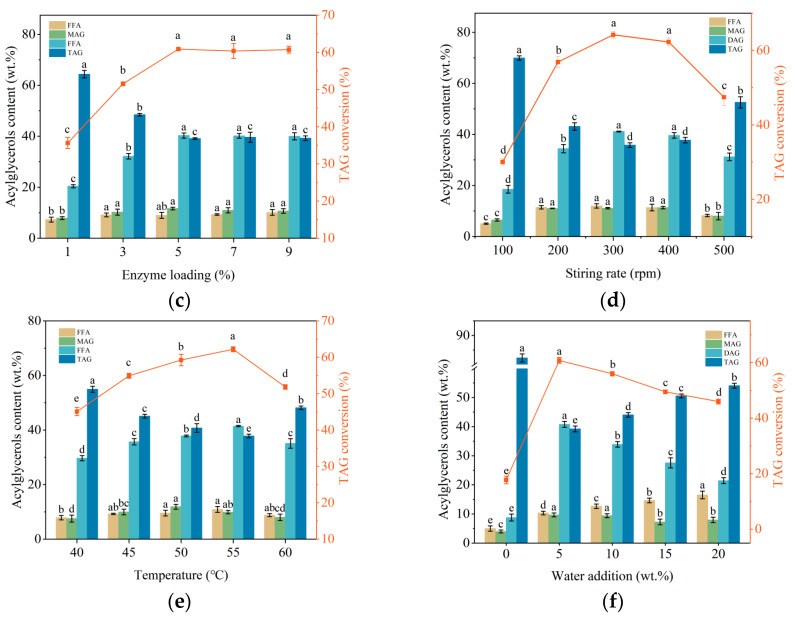
Selection of catalytic conditions for immobilized enzyme: (**a**) substrate molar ratio (glycerol/oil); (**b**) reaction time; (**c**) enzyme loading; (**d**) stirring speed; (**e**) reaction temperature; (**f**) and water addition.

The substrate molar ratio (glycerol/oil), reaction time, lipase loading, stirring speed, reaction temperature, and water addition were critical factors affecting the immobilized lipase’s catalytic efficiency in glycerolysis, as shown in Figure 4. In Figure 4a, it can be observed that with an increase in the substrate molar ratio (glycerol/oil), the DAG content initially rose and then declined, reaching a maximum when the molar ratio was 10:1. This was because an appropriate increase in glycerol enhanced the reaction rate by improving mass transfer efficiency, allowing more glycerol molecules to interact with oil molecules and promoting the reaction equilibrium towards MAG and DAG formation. However, excess glycerol may lead to the overproduction of side products, increasing the system’s viscosity and affecting mass transfer rates, thus reducing the reaction rate and enzyme activity. Conversely, low glycerol concentration may result in incomplete reactions and lower product yield due to insufficient glycerol to drive the reaction equilibrium. The impact of reaction time on catalytic efficiency is depicted in Figure 4b. As reaction time increased, the DAG content in the product reached its peak at 4 h and showed no significant change with further extension. Short reaction times resulted in incomplete reactions due to insufficient contact time between substrate molecules and lipases, leading to lower reaction rates. With prolonged reaction times, enzyme active sites became saturated, and the reaction reached dynamic equilibrium, causing no further increase in DAG content. Figure 4c illustrates the effect of lipase loading on glycerolysis efficiency, showing an initial increase followed by a plateau. An appropriate increase in lipase loading enhanced the reaction rate by providing more active sites, thus promoting substrate conversion. However, excessively high lipase loading yields limited increases in reaction rate and product yield due to substrate concentrations becoming a limiting factor. Conversely, low lipase loading results in insufficient active sites, leading to lower reaction rates and product yields. The influence of stirring speed on the catalytic efficiency of the immobilized lipase is shown in Figure 4d. As the stirring speed increased, the DAG content rose, reaching a peak at 300 rpm, and then declined. Higher stirring speeds enhanced mass transfer efficiency, allowing substrate molecules to reach the lipase’s active sites more quickly, thus increasing the reaction rate. However, excessive stirring speeds may cause mechanical shear forces that disrupt the immobilized lipase’s structure, leading to mechanical inactivation and reduced catalytic efficiency and product yield. Figure 4e demonstrates the effect of temperature on the catalytic efficiency of immobilized lipase. As the temperature increased, the DAG content rose due to enhanced molecular motion, which facilitated substrate interactions with the lipase’s active sites, thereby increasing the reaction rate. Beyond 55 °C, further temperature increases resulted in decreased catalytic efficiency due to lipase denaturation, which disrupted lipase structure and reduced catalytic activity. The impact of water addition on the reaction is shown in Figure 4f. With no water added, the reaction was incomplete, and the DAG content was only 22.12 ± 0.74%. As the water addition increased to 5 wt.%, the DAG content reached its maximum. Further increases in water addition caused a gradual decline in DAG content. Appropriate water addition enhances reaction rate and product yield by aiding substrate dissolution and maintaining lipase hydration, which preserves enzyme activity and stability. Excessive water dilutes the reactants, reduces the reaction rate, and may cause the partial deconstruction of the enzyme structure, reducing its catalytic activity. Conversely, insufficient water increases medium viscosity, limits substrate diffusion, and reduces enzyme activity.

To verify the applicability of TL-PEGDGE-LX, we selected seven common edible oils as substrates for the glycerolysis reaction under chosen conditions. The DAG content and TAG conversion rate of the products are presented in Table 2. As shown, the initial DAG content of all the substrates was low, ranging from 1.40 ± 0.01% to 2.95 ± 0.05%. After the glycerolysis catalyzed by the immobilized lipase, the DAG content in the products significantly increased, ranging between 32.24 ± 0.88% and 41.65 ± 0.13%, with the TAG conversion rate ranging between 47.97 ± 0.65% and 62.09 ± 0.97%. Notably, when corn oil was used as the substrate, the DAG content reached the highest value of 41.65 ± 0.13%. This indicated that the immobilized lipase exhibited broad applicability across different edible oils.

## 3. Materials and Methods

### 3.1. Materials

The resin LX-201A was provided by Xian Lanxiao Co., Ltd. (Xi’an, China). PEGDGE was purchased from Shanghai Maclin Biochemical Technology Co., Ltd. (Shanghai, China). Lipase CN-TL (from *Thermomyces lanuginosus*) was purchased from Vland Biotech Co., Ltd. (Qingdao, China).

### 3.2. Pretreatment of Resin

The resin was immersed in a 95% ethanol solution by volume for 24 h to achieve complete swelling. It was subsequently rinsed with deionized water until odorless. Following this, it was subjected to separate 3 h immersions in a 5 g/100 mL HCl and 5 g/100 mL NaOH solution. Subsequent to these treatments, continuous washing with deionized water was carried out until the effluent in the test tube was no longer turbid or exhibited any noticeable ethanol odor. The pretreated resin was sealed and stored in a refrigerator at 4 °C for future use.

### 3.3. Lipase Immobilization

Cross-linking was performed by shaking 5 mL of a 2% (w/v) PEGDGE with 0.5 g of resin at 250 rpm and 35 °C for 4 h. Subsequently, 5 mL of the lipase solution was introduced, and the reaction was allowed to proceed for an additional 4 h. The immobilized lipase was then obtained by filtering the mixture through a Buchner funnel, followed by three washes with a buffer solution and air drying at room temperature.

### 3.4. Determination of Enzyme Activity

The enzyme activity was evaluated using a procedure described previously [33]. Initially, a solution of polyvinyl alcohol (2 g/100 mL) and olive oil in a 3:1 ratio was blended at 5000 rpm for 6 min, with a 5 min interval between the two blending cycles. Next, 4 mL of the prepared olive oil emulsion was mixed with 5 mL of sodium phosphate buffer (100 mmol/L, pH 7.5) and heated in a water bath at 30 °C. Following this, 1 g/mL of lipase was added to the mixture and allowed to react for 15 min. The reaction was then stopped by adding 15 mL of 95% ethanol solution. The resultant solution was titrated with a 0.05 mol/L sodium hydroxide solution, using phenolphthalein as an indicator. A control sample was prepared in the same manner but without adding lipase. Enzyme activity, defined as the amount of lipase (1 g or 1 mL) that catalyzes the hydrolysis of the substrate to produce 1 µmol of fatty acid per min, was expressed in units (µ/g or µ/mL). The enzyme activity (µ/g or µ/mL) was determined using the following equation:

(1) $$X=\frac{(V_{1}-V_{0})\times c\times n\times 10^{3}}{t}$$

In this equation, V_1_ represents the volume (mL) of sodium hydroxide solution used to titrate the sample, while V_0_ denotes the volume (mL) of sodium hydroxide solution used for the control. The variable c indicates the concentration (mol/L) of sodium hydroxide, n stands for the ratio used to dilute the original enzyme solution to a concentration suitable for measurement, and t is the reaction duration (min).

### 3.5. Protein Content of Lipase

The protein content was quantified using the Kjeldahl method [34]. Initially, immobilized lipases were homogenized in sulfuric acid using a digestion system equipped with a fume neutralizer, then subjected to digestion at 420 °C. Following this process, the total nitrogen content was determined by titrating the resultant ammonium.

### 3.6. Analysis of the Acylglycerol Compositions

The acylglycerol compositions were analyzed using a gas chromatograph (GC, Agilent Technologies 7820A Series Network Gas Chromatograph, Agilent Technologies, Inc., Palo Alto, CA, U.S.A.) following the method described by Li et al. [35]. Initially, 50 mg of triacylglycerol (TAG) were dissolved in 5.0 mL of *n*-hexane, and the resulting solution was filtered through a membrane into a sample vial. The GC system was equipped with a DB-1ht capillary column (15 m × 0.25 mm × 0.1 μm) operating with a split ratio of 40:1. The column temperature was held at 50 °C for 1 min, then increased to 100 °C at a rate of 50 °C/min. The oven temperature was subsequently ramped up to 220 °C at 80 °C/min and further to 290 °C at 30 °C/min. Finally, the temperature was increased to 380 °C at a rate of 50 °C/min and held for 3 min. Nitrogen (N_2_) served as the carrier gas at a flow rate of 4.17 mL/min. The content of DAG was quantified using the area normalization method.

The conversion rate of TAG was calculated by comparing the mass difference of TAG before and after the reaction. The TAG conversion (%) was determined using the following equation:

(2) $$X=\frac{W_{1}-W_{0}}{W_{1}}\times 100\%$$

In this equation, W_1_ represents the initial TAG content (wt.%), while W_0_ denotes the residual TAG (wt.%) content post-reaction.

### 3.7. Isomer Analysis

The proportion of sn-1,3 Diacylglycerol (sn-1,3 DAG) and sn-1,2 Diacylglycerol (sn-1,2 DAG) isomers in the DAG was analyzed according to a previous method [20]. Pulsed Nuclear Magnetic Resonance Hydrogen Spectroscopy (^1^H-NMR, AVANCE III, Bruker Optics, Ltd., Milton, ON, Canada) was employed for detection, and the specific procedure is outlined as follows: approximately 10–20 mg of purified DAG samples was weighed and completely dissolved in 0.5 mL of CDCl_3_. Subsequently, the solution was transferred to an NMR tube for analysis. The parameters for ^1^H-NMR were as follows: acquisition time of 3.17 s, pulse width of 12.76 microseconds, spectral width of 10330.6 Hz, 80 scans, and a temperature of 25 °C.

### 3.8. Characterization of the Immobilized Lipase

The surface morphology of the resin and immobilized lipase was examined using scanning electron microscope. Before observation, all samples were coated with gold. The SEM analysis was performed at an accelerating voltage of 10 kV using an S-3700N scanning electron microscope (Hitachi Co. Ltd., Tokyo, Japan), with representative images selected for presentation.

TGA was performed on the support, free, and immobilized lipases. Approximately 5 mg of each sample was heated in N_2_ from 25 to 600 °C at a rate of 10 °C/min using a thermogram analyzer (Mettler Toledo, DE, USA).

The functional groups were analyzed with a FTIR spectrometer (Nicolet IS10, Waltham, MA, USA). Modifications were made to the method described by Kulkarni et al. [33]. Infrared spectra for LX-201A, lipase CN-TL, and TL-PEGDGE-LX were recorded over the range of 4000 to 400 cm^−1^, with samples dispersed in KBr pellets. Absorption peaks within the amide I region (1600–1700 cm^−1^) were identified. The obtained spectral bands were processed using Peakfit version 4.12, allowing for the determination of the lipase molecules’ secondary structure through a peak fitting analysis.

### 3.9. Statistical Analysis

The experiments were performed three times, and results are presented as mean ± standard deviation. Using SPSS 27 statistical software (SPSS Inc., Chicago, IL, USA), a one-way ANOVA was conducted for the statistical analysis. Duncan’s Multiple Range Test was applied to determine significance at *p* ≤ 0.05.

## 4. Conclusions

The immobilization conditions for the lipase were selected to maximize the DAG content in the glycerolysis reaction products. The selected conditions were pH 7.0, 35 °C, 2.0% cross-linking agent concentration, 4 h cross-linking time, 5 mg lipase per gram of support, and 4 h adsorption time. Enzymatic characterization revealed that the immobilized lipase TL-PEGDGE-LX exhibited enhanced pH stability, thermal stability, storage stability, and operational stability compared to the free lipase, and it possessed sn-1,3 specificity. Subsequently, the conditions for the immobilized lipase-catalyzed glycerolysis were analyzed. Under the selected conditions of a glycerol/flaxseed oil ratio of 1:10, lipase loading of 5%, water loading of 5%, temperature of 55 °C, reaction time of 4 h, and stirring speed of 300 rpm, the catalytic efficiency reached its peak. Common edible oils were then selected as substrates for the glycerolysis reaction, resulting in a DAG content in the products ranging from 32.24% to 39.76%, demonstrating the broad applicability of the immobilized lipase. By demonstrating the broad applicability of the immobilized lipase across various edible oils, the potential for DAG production under different substrates and reaction was confirmed, which is valuable in the food and health industries. Additionally, immobilization enhances the overall enzymatic properties of lipase and improves the sustainability and efficiency of industrial lipid production, making it more suitable for repetitive and large-scale industrial applications, highlighting its potential application prospects in the food and oil industries.

## Figures and Tables

**Table 1:** The content and proportion of sn-1, 2 DAG and sn-1, 3 DAG in the reaction products catalyzed by the immobilized lipase TL-PEGDGE-LX and the free lipase CN-TL with different kinds of oil as substrates ^1,2^.

	CN-TL			TL-PEGDGE-LX		
	sn-1,2 DAG	sn-1,3 DAG	sn-1,3 DAG/sn-1,2 DAG	sn-1,2 DAG	sn-1,3 DAG	sn-1,3 DAG/sn-1,2 DAG
**Olive Oil**	1.75 ± 0.12	8.25 ± 0.12	4.72 ± 0.25 ^a^	1.96 ± 0.12	8.04 ± 0.12	4.20 ± 0.23 ^b^
**Canola Oil**	2.72 ± 0.13	7.28 ± 0.13	2.69 ± 0.26 ^a^	2.77 ± 0.18	7.23 ± 0.18	2.62 ± 0.36 ^a^
**Peanut Oil**	2.34 ± 0.09	7.66 ± 0.09	3.34 ± 0.19 ^a^	2.33 ± 0.14	7.67 ± 0.14	3.30 ± 0.26 ^a^
**Soybean oil**	2.21 ± 0.15	7.79 ± 0.15	3.57 ± 0.28 ^a^	2.34 ± 0.23	7.66 ± 0.23	3.36 ± 0.44 ^a^
**Corn Oil**	2.09 ± 0.08	7.91 ± 0.08	3.78 ± 0.18 ^a^	2.18 ± 0.14	7.82 ± 0.14	3.60 ± 0.29 ^a^
**Palm Oil**	2.43 ± 0.15	7.57 ± 0.15	3.11 ± 0.21 ^a^	2.51 ± 0.11	7.49 ± 0.11	2.98 ± 0.16 ^a^
**Almond Oil**	2.67 ± 0.09	7.33 ± 0.09	2.74 ± 0.28 ^a^	2.69 ± 0.23	7.31 ± 0.23	2.71 ± 0.44 ^a^

^1^ Reaction condition: glycerol/flaxseed oil, 10:1 (mol/mol); water, 5% (wt/wt, based on substrates weight); enzyme, 5% (wt/wt, based on substrates weight); 50 °C, 4 h, 300 rpm/min. ^2^ The means ± standard deviation with different letters denote significant difference at *p* ≤ 0.05 (*n* = 3).

**Table 2:** The content of diacylglycerol (wt.%) and the conversion of triacylglycerol (%) in glycerolysis products were measured for different types of oils as substrates catalyzed by TL-PEGDGE-LX ^1^.

Sample	Olive Oil	Canola Oil	Peanut Oil	Corn Oil	Soybean Oil	Palm Oil	Almond Oil
**Initial DAG**	1.98 ± 0.11	2.01 ± 0.05	1.40 ± 0.01	2.38 ± 0.06	1.57 ± 0.09	2.95 ± 0.05	1.78 ± 0.11
**DAG**	38.98 ± 0.35	32.24 ± 0.88	39.76 ± 0.70	41.65 ± 0.13	34.57 ± 0.89	36.18 ± 1.07	37.31 ± 0.35
**TAG Conversion**	56.45 ± 0.86	47.97 ± 0.65	60.87 ± 1.12	62.09 ± 0.97	52.43 ± 0.54	55.86 ± 0.77	56.01 ± 0.86

^1^ Each value represents the mean ± SD (*n*  =  3).

## Data Availability

The data presented in this study are available on request from the corresponding author.
